# Effect of Neuromuscular Electrical Stimulation on Masseter Muscle Thickness and Maximal Bite Force among Healthy Community-Dwelling Persons Aged 65 Years and Older: A Randomized, Double Blind, Placebo-Controlled Study

**DOI:** 10.3390/ijerph17113783

**Published:** 2020-05-26

**Authors:** Moon-Young Chang, Gihyoun Lee, Young-Jin Jung, Ji-Su Park

**Affiliations:** 1 Department of Occupational Therapy, Inje University, Gimhae 50834, Korea; myot@inje.ac.kr; 2 Department of Physical and Rehabilitation Medicine, Center for Prevention and Rehabilitation, Heart Vascular Stroke Institute, Samsung Medical Center, Sungkyunkwan University School of Medicine, Seoul 06351, Korea; ghlee@skku.edu; 3 Department of Radiological Science, Health Sciences Division, Dongseo University, Busan 47011, Korea; microbme@dongseo.ac.kr; 4 Advanced Human Resource Development Project Group for Health Care in Aging Friendly Industry, Dongseo University, Busan 47011, Korea

**Keywords:** neuromuscular electrical stimulation, masseter muscle thickness, maximal bite force

## Abstract

Aim: This study investigated the effect of neuromuscular electrical stimulation (NMES) on masseter muscle thickness and maximal bite force among healthy community-dwelling elderly persons older than 65 years. Materials and methods: A total of 40 participants were randomly assigned to the experimental and placebo groups. In the experimental group, NMES was applied to both masseter muscles, and electrical signals were gradually increased until the participants felt a grabbing sensation (range 6.0–7.5 mA) in the masseter muscle. The placebo group, in contrast, underwent NMES in the same manner and procedure as the experimental group with less electrical intensity (0.5 mA). All interventions were administered five times a week for six weeks, 20 min per day. The outcomes were masseter muscle thickness assessed using ultrasound and maximal bite force using a bite force meter. The level of significance was set as *p* < 0.05. Results: The experimental group showed a significant increase in both masseter muscle thickness and maximal bite force as compared with the placebo group (*p* = 0.002 and 0.019, respectively). Moreover, the degree of change in the masseter muscle thickness and maximal bite force significantly increased in the experimental and placebo groups (*p* < 0.001, both). Conclusions: This study demonstrated that NMES could be an effective modality for increasing masseter muscle thickness and maximal bite force in healthy older adults.

## 1. Introduction

Masticatory muscles which are part of the stomatognathic system consist of the following four muscles: masseter, temporal, lateral pterygoid, and medial pterygoid muscles [1]. Among these muscles, the masseter muscle is the most powerful and plays a primary role in swallowing by closing the chin tightly during anterosuperior movement of the hyolaryngeal complex [2,3]. Masticatory muscles are primarily responsible for the mechanical breakdown of food and forming the bolus during the oral phase of the swallowing process [4]. In addition, muscle contraction aids saliva secretion inside the oral cavity through irritation of the parotid gland and mixing of food to make swallowing more efficient [5,6].

Aging directly affects oral function including masticatory ability, and poor mastication is a health problem among the elderly [7,8]. A nationwide survey in South Korea reported that, as of 2014, 54.6% of the elderly population experienced difficulties in performing daily activities due to swallowing problems caused by a decrease in the masticatory ability [9]. Skeletal muscle atrophy and weakness are common among the elderly due to the aging process, a phenomenon referred to as sarcopenia [10,11]. Weakening of the masseter muscles by sarcopenia makes it difficult to chew foods in the oral phase prior to swallowing [8,12]. This negatively affects the consumption of various types of food and leads to presbyphagia. Presbyphagia is an age-related form of dysphagia that refers to disordered swallowing caused by aging without a direct neurological cause [13,14,15]. Therefore, it is important to maintain and improve masticatory muscle strength and occlusal force among the elderly to make swallowing safe.

Masticatory muscles are skeletal muscles, and strengthening exercises are effective for inducing physiological changes such as muscle strength and thickness. Previous studies have reported that masticatory exercise with gum resulted in an increase in bite force in preschool children and healthy adults [16,17], and Nakagawa et al. [18] reported that chewing exercise was effective for increasing not only bite force but also saliva secretion. However, since masticatory muscle strengthening exercise using resistance requires a lot of physical effort, elderly people who have reduced physical ability due to sarcopenia can find it difficult to perform.

In contrast, neuromuscular electrical stimulation (NMES) is a safe, noninvasive, and inexpensive therapeutic method that can be applied without relying on the subject’s physical abilities. NMES causes evoked contractions of muscles through electrical stimulation, and therefore can be expected to improve motor function, as well as myophysiological changes (e.g., muscle thickening and strength) [19,20,21]. NMES has been applied to limb skeletal muscles, as well as swallow-related muscles (e.g., orbicularis oris and suprahyoid muscle), resulting in increased muscle thickness and improved motor function in the elderly and in patients with neurological disorders including stroke [22,23,24,25,26]. Recent studies have attempted to apply NMES to the masseter muscle. Therefore, increased muscle activation in the masseter muscle has been demonstrated, suggesting the potential for rehabilitation therapy [27,28]. Furthermore, Lee et al. [29] reported that NMES applied to the masseter muscle improved overall oral function, including chewing function, in the oral phase of patients with dysphagia after stroke. Nevertheless, the effect of NMES on masticatory muscles remains unclear. There is a paucity of related studies; previous studies measured the effect on muscle activation using surface electromyography and swallowing function using videofluoroscopic swallow studies. Thus, the effect of NMES on masseter muscle thickness and bite force is unknown. Therefore, the present study aimed to investigate the effect of NMES on masseter muscle thickness and bite force among healthy elderly subjects.

## 2. Materials and Methods

### 2.1. Study Design and Participants

**Figure 1:**
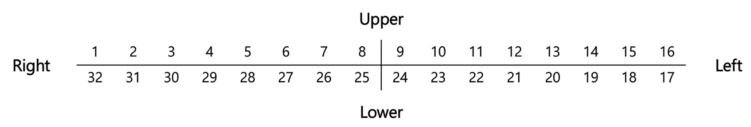
Universal numbering system (human teeth).

This study was designed as a two-group, randomized, double blind (participant and assessor), placebo-controlled study. This study was conducted at an elderly welfare facility in Korea. The present study enrolled 40 healthy elderly community-dwelling adults. The inclusion criteria were as follows: >65 years; no reported history of neurological diseases; no difficulty in chewing ability; normal swallowing and speaking ability; normal oropharyngeal structure; able to perform activities of daily living independently; ability to communicate and cooperate; and at least 24 teeth remaining (at least six teeth remaining at positions 1–8, at least six teeth remaining at positions 9–16, at least six teeth remaining at positions 17–24, or at least six teeth remaining at positions 25–32, each according to the universal numbering system) (Figure 1). The exclusion criteria were as follows: diagnosis of sarcopenia, implanted cardiac pacemaker, history of seizure or epilepsy, unstable vital signs, facial skin conditions, significant malocclusion or facial asymmetry, orofacial pain (including those caused by trigeminal neuropathy and toothache), and toothache due to periodontal disease.

### 2.2. Ethical Considerations

This study was approved by the Ethics Committee of the Seoul Medical Center in South Korea (2020-04-038-002). We described the complete details of this study to all participants and obtained their informed consent to participate.

### 2.3. Intervention

**Figure 2:**
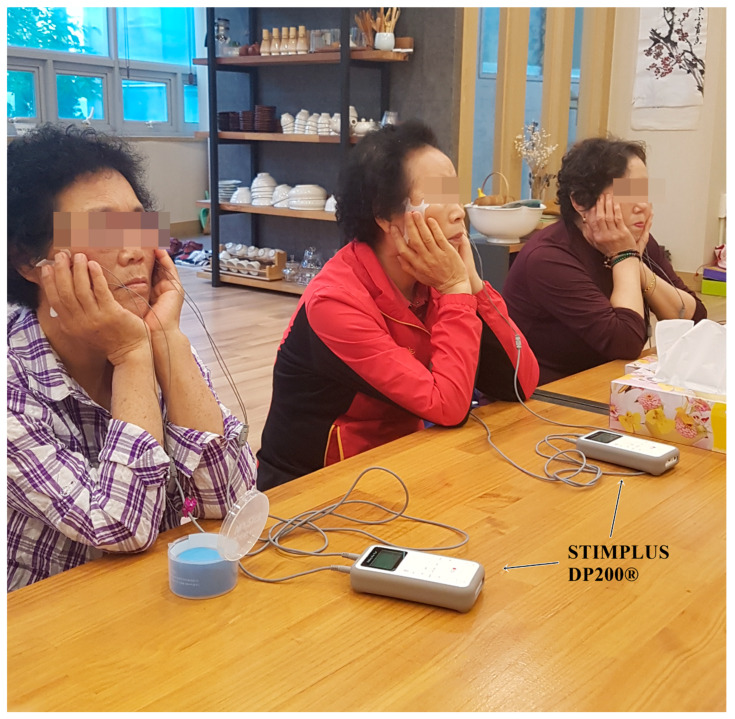
Neuromuscular electrical stimulation applied to both masseter muscles.

All participants were assigned randomly to the experimental (n = 20) or placebo group (n = 20) using blocked randomization. Allocation was concealed in sealed opaque envelopes. The experimental group received NMES on the bilateral masseter muscles. The NMES was applied using a STIMPLUS DP200^®^ (Cybermedic Corp., Iksan, Korea), which entails attaching a pair of electrodes to each masseter muscle. The electrode application area was shaven and cleansed with TENS Clean-Cote skin wipes. Then, attachment sites were marked using permanent ink. The NMES unit provided two channels of bipolar electrical stimulation at a 60 Hz pulse frequency and a pulse width of 500 μs. NMES was applied at motor level without reaching noxious levels to induce muscle contractions. The stimulation intensity increased gradually by 0.5 mA to the grabbing sensation level, that is, to an acceptable maximal level without pain, on the masseter muscles bilaterally (Figure 2). Among patients in the placebo group, NMES was applied in the same method with less stimulation intensity. The placebo group had the same exterior appearance as that of the real device used in the experimental group. However, only 0.5 mA of stimulus was applied such that there was little stimulation effect on the targeted muscles. All interventions were provided 5 times a week for 6 weeks, 20 min per day by an occupational therapist. The Consolidated Standards of Reporting Trials (CONSORT) statement (http://www.consort-statement.org/home/) was used as a framework [30].

### 2.4. Outcome Measurement

**Figure 3:**
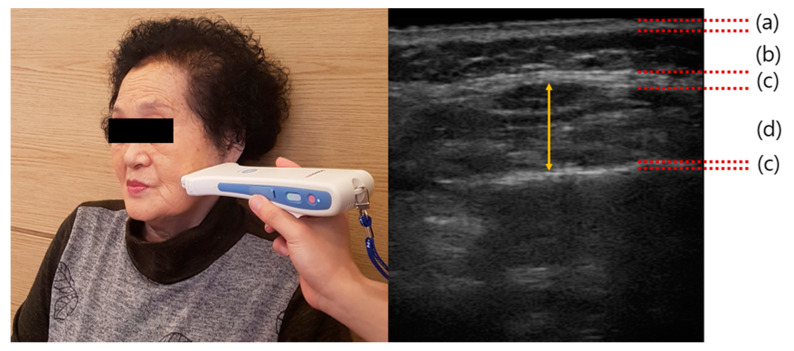
Measurement of masseter muscle thickness. Left panel, image recorded during the actual measurement of masseter muscle thickness and right panel, ultrasound image showing the anatomical structure of the masseter muscle. (**a**) Skin layer; (**b**) Fat layer; (**c**) Masseteric fascia; and (**d**) Masseter muscle.

The primary outcome of this study was the change in the thickness of the masseter muscle, which was measured using ultrasound. The thickness of the masseter muscle was evaluated using a portable ultrasound device (Sonon; Healcerion, Seoul, Korea). The participants were instructed to remain seated. The contraction of the left and right masseter muscles was assessed during clenching. The linear type transducer was set to a frequency of 10 MHz, 66 dB for all participants. The transducer was placed at the same angle as the line between the external auditory meatus and acanthion, and then moved down 2–3 cm to meet the mouth tail and the midpoint between the zygomatic arch and mandibular angle. The transducer was moved back 2–3 cm to meet the outer canthus level and masseter muscle level [31]. The thickness of the masseter muscle was determined at the thickest part of the image (Figure 3).

**Figure 4:**
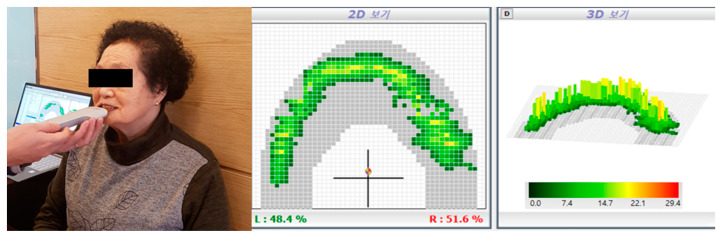
Measurement of the occlusal force. Left panel, image recorded during the actual measurement of the occlusal force; center panel, two-dimensional (2D) image of the measured data; right panel, 3D image of the measured data.

The secondary outcome of the present study was the occlusal force on both sides, which was measured using an Occluzer device (ACCURA; Demetec, Gyeonggi-do, South Korea) (Figure 4). The investigator who performed occulusal force assessment and analysis was blinded to all other parameters. The participants were instructed to sit on a chair in a relaxed, upright position, and then to bite the pressure-sensitive film (disposable pressure film, Gyeonggi-do, South Korea) as hard as possible. While in the seated position, the participants were instructed to place their incisor in the middle aspect of the bite sensor. The occlusal force was measured for 5 s, and the mean of three measurements was used for the analysis. The maximum occlusal force was expressed as an absolute value in newtons. All evaluations were performed by a blind radiological technician who did not interact with the participants.

### 2.5. Statistical Analysis

The descriptive statistics are presented as means with standard deviations. Participant characteristics were analyzed using SPSS Statistics version 20 (IBM Corp., Armonk, NY, USA). The Shapiro–Wilk test was used to check the normality of the outcome variables. To evaluate the training effects, the paired *t*-test was used to compare measures before and after the intervention in each group. The independent *t*-test was used to compare post-intervention values and changes in outcome measures between the two groups. The level of significance was set as *p* < 0.05. Analysis items with *p* < 0.05 were considered to be statistically significant. In addition, the effect sizes (Cohen *d*) of the changes in scores between the two groups were calculated. Effect sizes of 0.2, 0.5, and 0.8 represented small, moderate, and large effects, respectively.

## 3. Results

### 3.1. General Characteristics of the Participants

**Figure 5:**
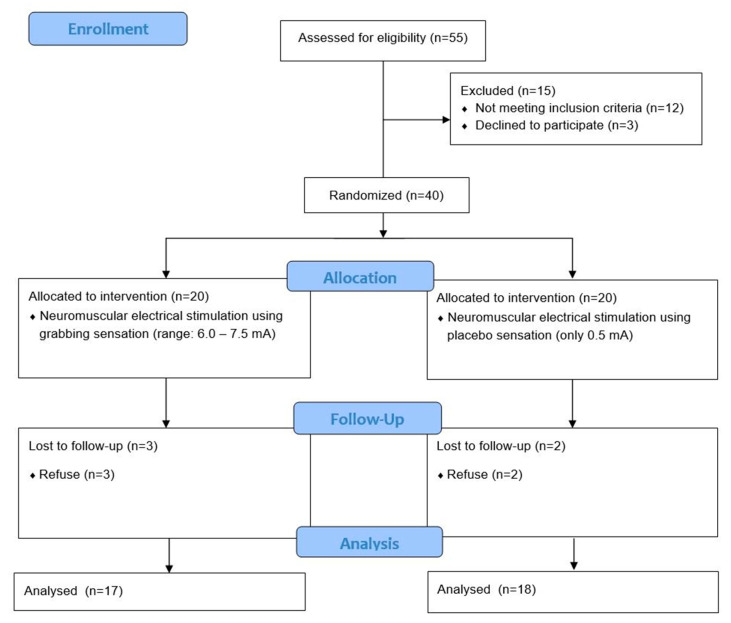
Flow chart of the trial.

Altogether, 40 participants were enrolled in this study. Three participants dropped out of the experimental group (due to refusal of the subjects), and two participants dropped out of the placebo group (due to refusal of the subjects). Therefore, the data from 35 participants were analyzed. Table 1 shows the general characteristics of the participants. The homogeneity tests for each item measured yielded no significant differences in baseline clinical and demographic data between the groups (*p* > 0.05). The flowchart of the study is shown in Figure 5.

### 3.2. Effect of Masseter Muscle Thickness

On the one hand, the masseter muscle thickness improved significantly in the experimental group following intervention from 7.36 ± 0.50 to 8.34 ± 0.61 (*p* = 0.001). On the other hand, the placebo group showed no significant improvement in the masseter muscle thickness from 7.60 ± 0.49 to 7.69 ± 0.45 (*p* = 0.087). In terms of the results of the comparison between the groups after intervention, the experimental group showed a significant improvement in masseter muscle thickness as compared with the placebo group (*p* = 0.002) (Table 2). Moreover, the amounts of change in muscle thickness were significantly higher in the experimental group than in the placebo group, with an average change of 0.98 ± 0.61 (experimental group), and 0.08 ± 0.18 (placebo group) in the masseter muscle thickness (*p* < 0.001) (Table 3).

### 3.3. Effect of Maximal Bite Force 

On the one hand, the maximal bite force improved significantly in the experimental group following intervention from 261.13 ± 9.84 to 275.20 ± 9.43 (*p* = 0.001). On the other hand, the placebo group showed no significant improvement in the maximal bite fore from 265.87 ± 9.97 to 266.60 ± 9.67 (*p* = 0.065). In terms of the results of comparison between the groups after intervention, the experimental group showed a significant improvement in maximal bite fore as compared with the placebo group (*p* = 0.019) (Table 2). Moreover, the amounts of change in muscle thickness were significantly higher in the experimental group than in the placebo group, with an average change of 11.07 ± 4.34 (experimental group), and 0.73 ± 1.43 (placebo group) in the maximal bite fore (*p* < 0.001) (Table 3).

### 3.4. Reported Side Effects

Adverse events were not reported during the study and at one month after the completion of the study.

## 4. Discussion

The present study investigated the effect of NMES on masseter thickness and maximal bite force among healthy elderly persons. The experimental group showed a significant increase in masseter muscle thickness and bite force as compared with the placebo group, indicating that NMES is effective at inducing masseter muscle thickening and bite force.

To the best of our knowledge, the present study is the first to investigate the effects of NMES on masseter muscle thickening and bite force among healthy elderly persons. NMES applied to skeletal muscle can yield positive effects. NMES refers to the induction of muscle contractions by depolarization of the nerve fibers using electrical stimulation [32,33]. Previous studies have reported that NMES is effective at increasing masseter muscle activation [27,28] and increased muscle activation reflects an increase in motor unit activation in the peripheral nervous system [34,35]. This indicates that the discharge rate of the motor unitsincreased or the number of recruited motor units increased, reflecting an increase in the strength of the muscle [36]. Therefore, repeated NMES application can cause activation of the target muscle and contribute to the increase in its thickness. In addition, muscle thickening has a direct effect on generating peak power through recruitment of more motor units. In previous studies, NMES has been applied to various skeletal muscles of the upper or lower limb [37,38,39]. NMES has been shown to induce thickening of skeletal muscles, including shoulder muscles [24], quadriceps [40], mid-thigh, and calf muscles [41], which is consistent with the results of the present study.

In particular, NMES can be expected to yield positive results among the elderly who are vulnerable to sarcopenia. NMES is a strong stimulus for motor unit recruitment of type II fibers and evokes a contraction [34,35,42,43]. Sarcopenia induces muscle weakness through atrophy and loss of muscle fibers, particularly affecting type II (fast) fibers [44,45,46]. Therefore, the application of NMES to improve muscle physiology and motor function in the elderly directly affected by sarcopenia would not only help induce myophysiological changes through stimulation of type II fibers, but also contribute to the prevention of muscle atrophy. Therefore, in this study, it was assumed that NMES would have a positive effect on masseter muscle thickness and would increase the bite force among community-dwelling elderly persons.

The present study confirmed that NMES could be a therapeutic modality to increase the masseter muscle thickness and maximal bite force among elderly persons. However, when applying this method, the intervention protocol (e.g., electrical intensity and schedule) must be carefully determined. Although the effectiveness of NMES depends on the application protocol, the optimal protocol is still not known. This study reported that the sixweek NMES protocol (electrical intensity, motor level, and schedule of five times a week for six weeks, 20 min per day) was effective for masseter muscle thickness and maximal bite force in the elderly. Therefore, a more effective and efficient protocol should be developed through modification of the protocol parameters, such as the duration of the intervention.

The present study had some limitations. First, the results of this study are not generalizable due to the small number of subjects. Second, the effect of NMES on the sensory level is unknown because the intensity of NMES was applied only to the motor level in this study. Therefore, these limitations should be addressed and the effects of NMES on oral function should be further investigated in studies conducted in the future.

## 5. Conclusions

This study demonstrated that NMES is effective for improving masseter muscle thickness and maximal bite force among healthy community-dwelling persons aged > 65 years. The results showed that NMES could improve the swallowing function and act as a therapeutic modality for improving masseter muscle thickness and maximal bite force.

## Figures and Tables

**Table 1:** General characteristics of the subjects.

	Experimental Group	Placebo Group
Number of subjects	17	18
Gender (male/female)	7/10	10/8
Age (year)	72.4 ± 4.1	73.3 ± 4.7
Height (cm)	166.2 ± 5.8	165.1 ± 7.5
Weight (kg)	64.2 ± 7.3	65.3 ± 6.5
Total number of teeth remaining	23.5 ± 2.2	23.2 ± 2.1
Masseter muscle thickness	7.36 ± 0.50	7.60 ± 0.49
Maximum occlusal force	261.13 ± 9.84	265.87 ± 9.97
Stimulation intensity (mA)	7.3 ± 3.5	0.5

**Table 2:** Changes of masseter muscle thickness and maximum occlusal force in parameters, before and after treatment.

	Experimental Group (*n* = 17)			Placebo Group (*n* = 18)			Intergroup *p*-Values
	Before	After	*p*-Value	Before	After	*p*-Value	
MMT (mm)	7.36 ± 0.50	8.34 ± 0.61	0.001 *	7.60 ± 0.49	7.69 ± 0.45	0.087	0.002 ^†^
MOF (newton)	261.13 ± 9.84	275.20 ± 9.43	0.001 *	265.87 ± 9.97	266.60 ± 9.67	0.065	0.019 ^†^

Mean ± standard deviation; MMT, masseter muscle thickness; MOF, maximum occlusal force; * *p* < 0.05 by paired *t*-test and ^†^ *p* < 0.05 by independent *t*-test.

**Table 3:** Comparison of amount of change in each group.

	Experimental Group	Placebo Group	Intergroup *p*-Value	Cohen’s *d* (Interpretation)
Δ MMT (mm)	0.98 ± 0.61	0.08 ± 0.18	<0.001 ^†^	2.6 (large effect)
Δ MOF (newton)	11.07 ± 4.34	0.73 ± 1.43	<0.001 ^†^	2.0 (large effect)

Mean ± standard deviation; MMT, masseter muscle thickness; MOF, maximum occlusal force ^†^ *p* < 0.05 by independent *t*-test.
